# Factors affecting health-seeking behavior in sports climbers in Metro Manila: a cross-sectional study

**DOI:** 10.3389/fspor.2025.1514827

**Published:** 2025-02-24

**Authors:** Danyelle Jay T. Cruz, Jan-Tyrone C. Cabrera

**Affiliations:** Department of Physical and Rehabilitation Medicine, Ospital ng Makati, Makati, Philippines

**Keywords:** sport climbing, factors affecting health-seeking behavior, Action-intention subscale, bouldering, climbing injuries

## Abstract

**Introduction:**

In the Philippines, studies on athletes are hard to come by when compared to those done internationally, even more so on newer Olympic sports such as Sports climbing. Furthermore, there is a lack of internationally published research on professional health-seeking behavior for the physical conditions of Filipino athletes. Thus, the objective of the study was to investigate the characteristics of sports climbers in Metro Manila that were associated with better health-seeking behavior.

**Materials and methods:**

An analytical cross-sectional study design was done using an online interview-assisted questionnaire. The Action-intention subscale of the Attitudes Towards Medical Help-seeking Scale was used to measure health-seeking behavior in the study population. Other data gathered includes the population characteristics, injury characteristics, and social-environmental characteristics of sports climbers in Metro Manila.

**Results:**

A total of 125 participants were interviewed, majority of whom were young adults and middle aged (72.8%), college graduates (67.2%), working full time (54.4%), middle class (22.4%), with slightly more males (49.6%). None of the social-environmental factors were found to be significant (*p* > 0.05). Trust in websites was found to be the only significant personal factor (2.99 ± 1.00, *p* < 0.05). Among the injury factors studied, the location of pain at the hip/pelvis and knee was found to be significant (*p* < 0.05).

**Discussion:**

Climbers in Metro Manila have a decreased tendency to engage in health-seeking behavior. Because of this, sports climbers in Metro Manila may be a vulnerable group in terms of availability of health care systems specific to the sport, accessibility of knowledge for self-diagnosis and management, and data enough present in the Philippines to create guidelines and protocols for climbing injuries. Better monitoring of information on websites is needed. Healthcare providers should also improve the way they approach and deal with sport climbing injuries.

## 1 Introduction

Climbing has become an increasingly popular sport with its world debut in the recent 2020 Tokyo Olympics (1). Along with its popularity and appeal as a sport, climbing comes with its own risks. A study conducted in 2009 on the Swedish Climbing Association showed that climbers received 4.2 injuries for every 1,000 hours of participation in activity, with overuse injuries accounting for 93% (2). In a study done on rock climbers of all levels in the United States, among injuries of the upper extremity injuries of the hand were the most common (3). Since 1996, researchers have known in terms of risk of injury there is no difference in rate of injuries when comparing indoor gym climbers and outdoor rock climbers (4). An even earlier study done in 1990 found that climbing hand injuries were specifically caused by the dynamic movements and strain placed on fingers from gripping small holds (5). With increased time spent on the activity, sports climbers tend to experience more chronic injuries (6). Other studies have found that lower climbing levels, the tendency to select more difficult routes, riskier climbing styles (i.e., boulder and lead climbing), and age have been associated with more hand and upper extremity injuries (7).

A study done on health-seeking behavior in the 2005 World Championships was alarming as it has been found that young climbers delayed reporting joint pain, continued training hard, and ignored medical advice (8). In 2017, research done on climbers with varied proficiencies showed that 81% of those studied chose not to seek professional help and 61% sought information from other sources—social media, specialty magazines, and websites (9). One problem seen was that while climbers did receive helpful information on self-management, a majority did not possess the tools to accurately self-diagnose (9). However, when provided with a presumably capable health care provider professional sports climbers chose to seek professional help (10).

In the United States, the health-seeking behavior of athletes in general are determined by disparity in resources among the socioeconomic groups (e.g., access to employment, transportation, safety of the physical environment, income), and cultural differences among populations when comparing race, class, gender expression, and sexual orientation (11). In school-aged athletes, an additional barrier to health-seeking is the lack of expert medical personnel available (12).

Likewise, in the Philippines, the healthcare system faces many challenges, namely the lack of funding, an overburdened health-care workforce, and the unequal allocation of resources with majority distributed in the capital region and its surrounding areas (13, 14). Because of this, health-seeking behavior in Filipinos is still strongly linked to both individual factors such as socioeconomic status and household income and external factors such as regional poverty levels and geographical location (15). Reliance on alternative sources of health information such as traditional healers (i.e., *albularyo, hilot*)*,* and suggestions from neighbors, friends, and family before seeking consultation with a physician is also common practice in the Philippines (15).

This study sought to find the facets that guided sports climber's decision making in relation to professional health-seeking behavior in order to possibly improve this behavior in climbers with hand injuries and their quality of life. No studies on the health-seeking behavior of the Philippine sports climbing community have been published internationally to date. Data taken from this research may help to establish benchmarks for Filipino climbers. Results of this study may help to facilitate best practices in the diagnosis and management of Sports climbers. From this study, specific public health infrastructure may be suggested to better improve the health-seeking behavior of climbers, as well as to provide possible interventions delivered to climbers at gyms and outdoors.

The primary objective of this study was to investigate the characteristics of sports climbers in Metro Manila that were associated with better health-seeking behavior. Specifically, this paper further sought to do the following: (1) To determine the demographic profile of sports climbers with hand injuries training in Metro Manila, (2) To determine what factors (e.g., disease factors, personal factors, social-environmental) have the highest association with willingness to engage in future health-seeking behavior as documented by scores obtained on the Attitude-Intention subscale of the Attitudes Towards Medical Help-seeking Scale.

## 2 Materials and methods

### 2.1 Study design

**Figure 1:**
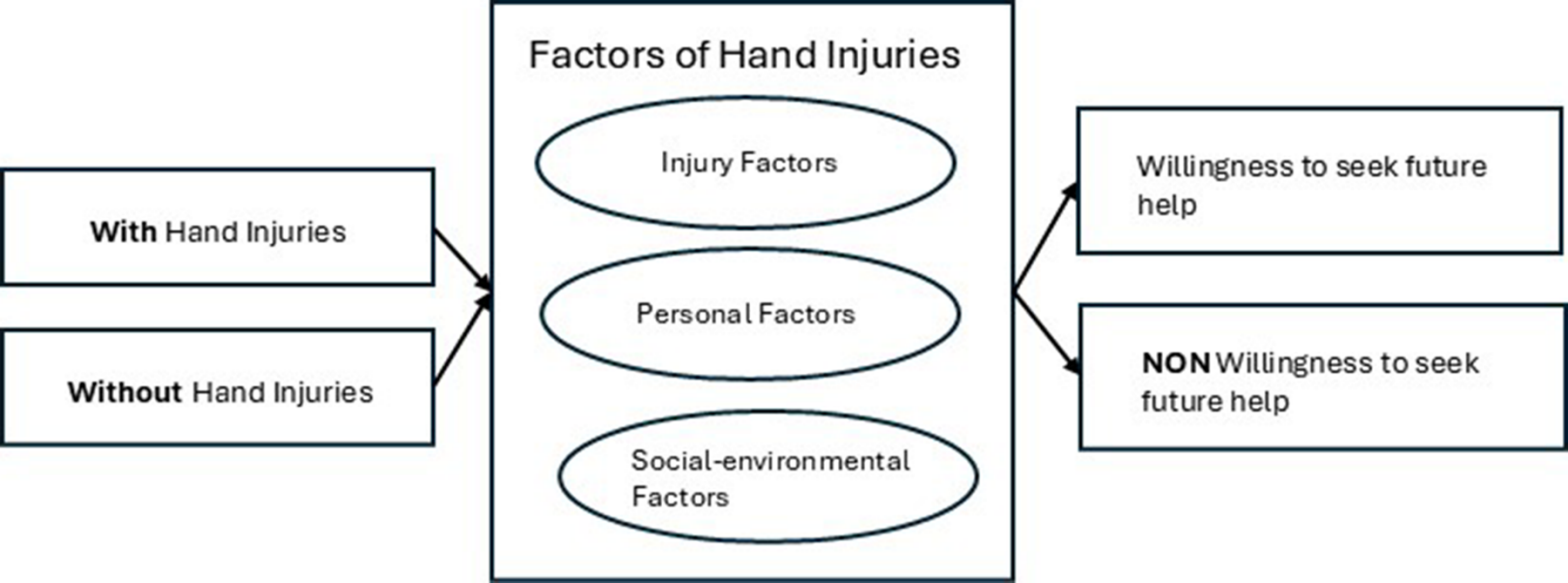
Conceptual design.

This study made use of an analytical cross sectional study design (See Figure 1).

### 2.2 Operational definitions

#### 2.2.1 Injuries and pain

Injuries referred to in this study were defined as musculoskeletal conditions which included any damage to the joints, soft tissues (i.e., muscles, tendon, nerve, ligaments, and vessels), or bone (16). Hand injuries were defined as any injuries occurring distal to the wrist, while upper extremity injuries include any injuries from the course of shoulder to the hand.

Pain in this study referred to musculoskeletal pain caused injuries to the musculoskeletal system (16, 17). Musculoskeletal pain is acute when its duration is less than three months and becomes chronic when its duration exceeds three months (17).

#### 2.2.2 Sports climbing

In this study, both disciplines of bouldering and lead climbing were included and referred to as “Sports climbing”. In bouldering, climbers without rope or harness aim to complete a set of routes called “problems” on walls no higher than 4.5 meters (18). Lead climbers are equipped with both rope and harness and aim to complete routes on walls with heights reaching nine meters (18). In the Olympic format, both lead climbing and boulder make use of a point system assigned to the highest part of the routes reached (19). Another style of climbing was also included—top rope—where climbers are anchored at the top of the route differentiating it from lead climbing. Unlike the other two events, in Speed climbing, two climbers compete by racing on the exact same route side by side for the fastest time (20).

In the Philippines, lead climbing routes are integrated into top rope facilities; therefore, the proponents of this study combined both lead climbing and top rope climbing into the term “Route climbing”; Furthermore, because speed climbing facilities are currently unavailable in the Philippines, it was not included in this study. In sports climbing, routes and problems are graded according to different metrics (e.g., technique, strength required). In this study, climbers were categorized according to the level of experience. For bouldering, climbers were placed in groups according to the Fontainebleau grading system (i.e., Recreational: 4 to 5+, Intermediate: 6A to 6C+, Experienced: 7A to 7C, Elite: 7C+ to 8A+, International Elite: 8b and above). Route climbers were ranked based on the French climbing grade reached (i.e., Recreational: 4 to 6b, Intermediate: 6b+ to 7a+, Experienced: 7b to 8b, Elite: 8b+ to 8c+, International elite: 9a and above) (21).

### 2.3 Study population

The eligibility criteria for participants included: (1) participants currently in Metro Manila, (2) within the ages of 18 to 60 years old, (3) having engaged in sports climbing within the past year (2022–2023), (4) with no minimum or maximum grade of climbing achieved using the French system, (5) with any experience level, (6) practicing bouldering or route climbing.

For the exclusion criteria, this study did not include participants who engaged in other climbing disciplines: trad climbing, deep water climbing, ice climbing, and free soloing. Participants with comorbidities predisposed to hand injuries were also excluded; as well as, patients who received injuries outside of climbing-related sporting activities and comorbidities that increase the risk for musculoskeletal injuries.

### 2.4 Outcome

The primary outcome to be studied in this paper is the Action-intention subscale of the Attitudes Towards Medical Help-seeking Scale, measuring health-seeking behavior, which consists of 12 factor-analyzed items, to be answered in a four-point Likert scale (3-0, agree-disagree) (22). The Action-intention subscale shows good predictive validity, with higher scores obtained meaning the more likely a person will seek medical advice (*p* = 0.001, *p* = 0.050) (22, 23). The Action-intention subscale also displays good internal consistency (*r* = 0.86, *r* = 0.82) and test-retest reliability (*r* = 0.84, *r* = 0.85) (22, 23). For the data analysis in this study, arbitrary categories were made with 0–12 (Low Likelihood), 13–24 (Intermediate Likelihood), and 26–30 (High Likelihood). See Supplementary Table 1.

### 2.5 Independent variables

The injury factors (i.e., number, location, kind of injury, chronicity, comorbid injuries) were compared to the Action-intention subscale. Personal factors (i.e., prior health-seeking behavior experience and preference) were correlated to the scores taken from the Action-intention subscale. Lasty, the effect of social-environmental factors (i.e., age, sex, climbing experience, other activities) on the Action-intention subscale was studied.

### 2.6 Data collection

Study participants were recruited using convenience and snowball sampling methods. Participants were enlisted through an online post on social media and communication platforms- Facebook, Viber, and Telegram groups. Messages and physical posters were sent to various climbing groups in universities, colleges, and climbing gyms.

### 2.7 Questionnaire

An online interview-assisted questionnaire taken from the study by Grønhaug (6) was sent to study participants. The following data was gathered: Population characteristics (i.e., age, sex, the number of hours and days per week engaged in sports climbing (combined bouldering and route climbing), French and Fontainebleau climbing grades, if the participants mainly practiced bouldering or route climbing—encompassing both lead and top rope), Injury characteristics (i.e., injuries, severity of pain, cause of injury, chronicity), and Primary and Secondary Outcomes (i.e., management done, sources of information, kind of professional advice sought—including kind of physician or health-care provider, use of common preventive strategies among climbers).

### 2.8 Pilot testing

A pilot study was conducted where a total of 60 climbers, with an equal number of males and females, mostly college graduates, full time employees, in the middle class, with route climbers with varying levels of experience, from different gyms within Metro Manila were recruited. The interview-assisted questionnaire was evaluated for the clarity of instructions, any ambiguous terminology, and technical errors. Participants’ understanding of each item, as well as feedback was collected during and after answering the survey. No items were omitted from the survey. Technical medical terms were defined. However, one item was added to screen for the exclusion criteria “Do you have any known DIAGNOSED (by a medical doctor) comorbidities illnesses diseases requiring medication, lifestyle change or daily management (Hypertension High blood diseases, Lung diseases (i.e., asthma, COPD), Diabetes, Rheumatoid Tendon and Joint diseases from birth or inherited (e.g., Marfan, Rheumatoid arthritis, Gout)?”.

### 2.9 Sample size

A calculated sample size of 100 participants was obtained using a 95% confidence level, 0.10 margin of error. The sample size was calculated using the average number of active members per month within a six-month period, taken from a climbing gym located in Metro Manila.

### 2.10 Statistical analysis

The Chi square test of association was used to compare climbers with hand injuries and those without and to determine factors that affect attitude. Descriptive statistics (i.e., mean and standard deviation) and frequency and percentage were also computed. Statistical analysis was done using SPSS 28.0 for Windows with a confidence interval of 95% (*p* < 0.05).

## 3 Results

### 3.1 Socio- demographic characteristics

A total of 125 participants were interviewed for this study, a majority of whom experienced hand injuries (75.2%). The age range of the respondents are as follows: 16–30 years old (72.8%), 31–40 years old (20.8%), and 41–50 years old (6.4%). For gender, there were more male participants (49.6%) vs. female participants (47.2%), while 3.2% identified as genderqueer or gender non-conforming. In terms of educational background, many of the participants were college graduates (67.2%) followed by those with postgraduate degrees (13.6%), high school graduates (8%), some college or no degree held (7.8%), and the remaining four percent comprised of those who entered vocational school, elementary school graduates, and no formal schooling. When it comes to occupation, the largest group was compromised of full-time employees (54.4%), followed by freelance workers or self-employed, (17.6%) and lastly, students (15.2%). There were more participants (22.4%) with an estimated average monthly income ranging from ₱19,041 to ₱38,080 compared to other groups. None of the previously mentioned demographic factors (i.e., age, gender, educational attainment, occupation status and income) were statistically significantly associated with the occurrence of hand injuries (*p* > 0.05). There were no statistical differences found between groups in all demographic data acquired. See Supplementary Table 2.

### 3.2 Social-Environmental factors

Supplementary Table 3.1 shows the results of the Action-intention, where a higher score shows increased willingness of participants to seek help in the future. Only three of those who participated (2.4%) had a score of 0–12, while 65 of them (52%) obtained a score of 13–24. The remainder of participants (*n* = 57, 45.6%) belong to the group with the highest score of 25–36. This implies that those who are more willing to seek future help (25–36) is slightly lower (45.6%) as compared to the combined score of 0–12 and 13–24 (54.4%). Generally, results show no conclusive difference between those groups, implying undecidedness of whether to seek future help or not. Results also show that age, gender, highest educational attainment, occupational status, and their estimated average monthly income are not statistically significantly associated with their action-intention score (*p* > 0.05).

### 3.3 Personal factors

Results in Supplementary Table 4 show that the most common training activity is climbing (95.2%). The study also found out that most participants practiced bouldering (62.4%) compared to route climbing (37.6%). The most common level of bouldering climbing performance by the respondents was Intermediate: French 6A to 6C+ V3–V5 (48.7%), followed by Experienced: French 7A–7C V6–V9 (29.5%). In terms of route climbing, the most frequent level reached was Intermediate: French 6b + - 7a + (55.3%) while the remaining respondents were Recreational climbers: French ≤6b (42.6%). 32.8% of participants spent an average of four to seven hours per week climbing followed by 24% of those who climbed for eight to ten hours per week. For participants who did not have climbing as their main discipline, weight training (53.6%) and running (27.2%) were the most common sports activities they engaged in. More participants spent four to seven hours per week engaging in other sports (40.8%) than those who spent only one to three hours (32.8%) of cross-training per week. Majority of the respondents have less than a year of climbing experience (38.4%), while 35.2% have experience of one to two years, while the rest have three years or more of climbing experience (26.4%). These variables are not statistically significantly associated with the Action-intention subscale score (*p* > 0.05).

Most respondents reported “never” using the following healthcare providers: Physicians (40.8%), Physical Therapists (41.6%), Sports Medicine Specialists (43.2%), Manual Therapists (71.2%), Other health professionals (56.0%). Both climbing coaches and non-climbing coaches were also “never” used as healthcare providers (51.2% and 56.0%). Participants preferred to perform self-diagnosis (25.6%) or consulted with a friend “occasionally” (30.4%). Websites were used as a source for healthcare “some of the time” (28%) while books were “never” used (54.4%). The frequency of use among these sources was not statistically significantly associated with the Action-intention subscale score (*p* > 0.05).

Sports medicine specialists obtained the highest level of trust from participants with 61.6% trusting them “all the time”, compared to Physical therapists (55.2%), Physicians (35.2%), Manual therapists (12.8%). Sources of healthcare that were trusted “occasionally” were Non-climbing coaches (30.4%) and the Opinion of peers (42.4%). Both Climbing coaches and Non-PT Manual therapist were “never” trusted by participants (40.8% and 25.6% respectively). In terms of trusting themselves, 28.8% of participants responded with “occasionally”. Both websites and books were trusted “occasionally” (43.2% and 32.8% respectively). Among the respondents’ level of trust of healthcare sources, only trust in websites were shown to be statistically significantly associated with the Action-intention subscale score (*p* < 0.05).

Majority of participants (40%) “neither agree or disagree” on whether to trust climbers with experience of a similar pain or injury more than health providers. However, participants expressed a preference for seeing a healthcare provider who is also a climber even if they were to travel an additional hour (43.2%). Majority of participants (87.5%) of those injured did not seek a healthcare provider because they thought “the injury was not serious enough”. Responses to the earlier statements were also not statistically significantly associated with the Action-intention subscale scale (*p* > 0.05).

### 3.4 Injury factors

In terms of injury factors, the presence of climbing injuries within the last year was not significantly associated with the action-intention subscale (*p* = 0.8). The most common location of pain reported among participants in the upper extremities was in the fingers (46.4%), while the most common location in the lower extremities was in the foot ankle (24.0%). The most common location of injury in the upper extremities was at the fingers (12.8%) while for the lower extremities were at the ankle/foot and hip/pelvis. The duration of these injuries usually lasted 28 or more days (65.9%), however, this was not significantly associated with the action-intention scale (*p* = 0.79). Injuries were mostly self-diagnosed (34.1%), while 29.5% went to a sports medicine specialist (29.5%). Majority of participants “trained at a reduced level” as a result of their injuries (38.6%). Among the injury factors mentioned prior, the presence of pain in the knee and hip pelvis were statistically significantly associated with lower Action-intention subscale scores (0–12) (*p* < 0.05).

## 4 Discussion

The proponents of this study have found that among the personal factors, regardless of socio-demographic profile, only those who trust websites as a source of information were the most likely to seek consultation. This finding is consistent with research done in 2019 by Weaver et al., which showed that internet medical information users reported greater general health care use (24). As early as 2007 in Europe, internet users were found to be more likely to decide to see a doctor and use the internet in preparing for an appointment (25). Furthermore, Shneyderman et al., found that individuals who seek out health information online are also more likely to adhere to illness screening (26). On the other hand, the opposite was also found to be true—“heavy users of health-care services” were almost twice as likely to use the internet to seek health information (27). This suggests that climbers who utilize online health services tend to exhibit more proactive health-seeking behaviors.

In terms of injury factors, only those who have experienced hip/pelvic and knee pain were more likely to seek professional help. One reason could be that patients who have chronic lower extremity injury have greater disability reported more than pain, which was found to be a decisive factor in health-seeking (28). One potential explanation for why lower limb injuries proved to be more of a concern for athletes compared to upper limb injuries could be attributed to their tendency to inaccurately gauge the seriousness of injuries and resultant decisions in seeking medical advice (29). This is highlighted by research done comparing health-seeking and non-health-seeking groups that showed no difference in location of pain but rather with their personal experiences in healthcare and being able to self-manage injury (30).

### 4.1 Sociodemographic characteristics of climbers

Looking at the results of this study, social-environmental factors were found to have no influence on health-seeking behavior. In this study, we found that the majority of those who answered the questionnaire were young adults and middle-aged persons, college graduates, working full time, who were part of the middle class, with slightly more males than females. The demographic profile in this study closely matched a study done on rock climbers in a developing country which concluded that while males are more likely to seek adventurous past times, females are also gaining interest in the sport (31). These researchers also found that climbers of that area were college graduate post-graduate degree holder professionals with the income to match (31). Compared to this, climbers in Norway interviewed in the study by Grønhaug were composed mostly of males, between the ages of 26–40 years old and have climbing experience of more than three years, while climbing at the intermediate level (6b+ to 7a+) (6).

### 4.2 Factors affecting health-seeking behavior

#### 4.2.1 Factors that improve health-seeking behavior

##### 4.2.1.1 Higher education and socioeconomic Status

Despite lacking in statistical significance, several factors have shown higher scores on the Action-Intention Subscale. As mentioned earlier, one of these factors is the demographic profile of climbers. In an earlier study done in Germany by Nölke, Mensing, Krämer, and Hornberg, education and occupational status was closely related to health-seeking behavior online (27). Multiple studies have shown that individuals with higher educational backgrounds were found to have better understanding, higher frequency of use, and easier access to online health sources (32, 33). A potential explanation is that adult athletes with higher socioeconomic status often possess greater health literacy, which increases their likelihood for health-seeking (34) Furthermore, Timpka et al, notes that athletes belonging to the upper social classes already have the available resources for regular consultation (34). This implies that while not directly influencing health-seeking behaviors, the social-environmental factors found surrounding climbers in this study align with profiles observed in other studies, which show a higher propensity for engagement in general healthcare practices. Belonging to a lower income bracket decreases the likelihood of seeking a professional medical opinion compared to those in higher income brackets.

##### 4.2.1.2 Experience and time spent on sports climbing

Personal factors that may increase the probability that climbers will seek help include more time spent on climbing and climbing higher performances in bouldering and route climbing. Time spent on non-climbing training activities also has the same effect. Findings seen in a study done on professional athletes in the International Federation of Sports Climbing showed that all respondents engaged in health-seeking behavior (10). A possible reason for this is that experienced athletes who also have greater exposure to injuries are also more likely to seek injury prevention methods compared to athletes with less experience or without injury exposure (35).

It can be inferred that the more experienced a climber becomes, the more likely they are to engage in self-care practices including using professional healthcare services.

##### 4.2.1.3 Healthcare provider preference

Another factor to consider is the preference for a healthcare provider who shares the same interest in climbing. Additionally, the likelihood of seeking professional help is heightened with previous experience with a sports medicine specialist. Findings are similar to a study by Piper et al., where climbers sought professional healthcare if they knew a trusted provider (9). Similarly, professional sports climbers sought consult from their health care providers afforded to them especially when there was increased perceived usefulness (10). In a study done on subjects with chronic pain, participants who had a previous physician consult within the past year rated the overall usefulness of professional health care as higher than those who had not consulted (30). This suggests that climbers are more inclined to obtain professional help if they see it as beneficial to their needs.

##### 4.2.1.4 Chronicity and severity of injuries

The presence of an injury for 28 days or more also increased the likelihood of health-seeking. In an earlier study, rock climbers were found to seek help when self-treatments were not working, and with worsening injuries through time (9). Climbers were also less likely to be able to accurately assess their own chronic injuries (36). A cohort study on chronic musculoskeletal pain also displayed similar results (37). In a general survey done on musculoskeletal injuries in Australia, it was found that across age groups as the duration and severity of pain increased participants were more willing to go out of their way to get help (38). Despite being the most common location of injuries, hand and finger injuries were considered as minor compared to lower extremity injuries which were often managed by a healthcare professional (39). Findings in a focus group discussion showed that athletes are more likely to seek different methods of self-treatment prior to seeking a medical diagnosis (26) which may explain why athletes seek consultation sometime after the injury has happened.

One more factor that displayed higher scores was if athletes completely stopped climbing or trained at a lower-level post-injury. In 2017, Piper et al., also previously reported that rock climbers consulted with physicians when they recognized that their injuries needed urgent care (9). Other studies have shown that rock climbers who sought professional medical advice had worse injuries and disability (40). This implies that climbers engage in health-seeking behavior only when they acknowledge the severity of their injury.

#### 4.2.2 Factors that worsen health-seeking behavior

##### 4.2.2.1 Younger age groups

Some factors demonstrated lower scores on the Action-Intention Subscale, even though they did not reach statistical significance. Conversely, regarding social-environmental aspects, individuals under the age of 30 exhibited a lower inclination to seek assistance compared to those aged 30 and above. Studies have found that younger age groups prefer to seek health information from alternative means such as media outlets compared to health professionals (38).

##### 4.2.2.2 Male gender identity

Compared to females, males also have a lower likelihood of seeking consultation. One contributing factor may be that women exhibit more positive attitudes toward seeking medical assistance compared to men (37, 41). This means that, given the same injuries as their male counterparts, female climbers are more inclined to seek assistance from professionals.

##### 4.2.2.3 Lack of experience with coaches and Low trust in healthcare professionals

As mentioned earlier, time spent climbing is a relevant factor, where less time spent on climbing means less likelihood of seeking help. Having no previous experience with professional medical workers, as well as climbing and non-climbing coaches, also showed a very low likelihood that climbers would get help, these included. The tendency for climbers to self-diagnose injuries also contributes to the preference for not seeking professional help. Furthermore, having a low level of trust for healthcare professionals increased the chances of not seeking professional help.

### 4.3 The sports climbing population in Metro Manila

One probable explanation for why most of the findings in this study did not reach statistical significance is that the climbing community in Metro Manila is both homogenous and emergent where decisions about health-seeking behavior are easily impacted by social influences. This was seen in a previous study done on upper extremity injuries in climbers where climbers showed a preference to seek health care information from peers with low perceived usefulness of healthcare professionals (15). Another element to consider is that when given a population with decreased behavioral variability the attitude to behavior correlation of the Action-intention subscale shows weaker reliability (22).

Climbers in Metro Manila showed the same health-seeking behavior as many Filipinos studied with reliance on other sources for health information (e.g., traditional healers, peers) (15). Like the general Filipino population, climbers were also more likely to seek help given greater household incomes and were less likely to seek help with lower education and socioeconomic status (15). Sports climbers were not immune to the weaknesses of the current Philippine health-care system.

This paper has shown that sports climbers studied have similar health-seeking habits when compared to other athletes. In one study done on French athletics, track and field athletes who have received at least one injury are also likely to adhere to injury reduction (35). Like the participants in this study, volleyball players were also more inclined towards physicians who are familiar with their sport and popular within their peer group (42). Stadden has found that across various collegiate sports, namely wrestling, basketball, swimming, and hockey, athletes had the tendency to first self-manage their chronic pain and injuries then later seek consultation when their performance has been affected (43). In the same study, young male athletes, similar to the population in our study, were also less likely to seek consultation (43). Meanwhile, several factors were found linked to better outcomes and adherence to health-care programs such as physical rehabilitation—perceived usefulness, higher level of sport performance, and social influence (43). This means that sports climbers, in terms of factors affecting help-seeking, were similar to other athletes.

## 5 Limitations and recommendations

One limitation of this study is that injuries are self-reported and lack medical examination. Although the questionnaire has been pilot tested, and use of the survey was interview-assisted there is no guarantee that climbers were able to accurately diagnose their own injuries in part due to recall bias. Furthermore, the study included a majority of participants who have climbed for less than one year and were limited to studying climbers within Metro Manila. Lastly, due to the low validity of the Action-Intention subscale in a group with less behavioral variation, the health-seeking behaviors were possibly underestimated.

The proponents of this study suggest the use of alternate study design and more appropriate participant sampling strategies. Future researchers may benefit from limiting their study to climbers with more experience. Another suggestion is to increase the scope of future studies to a national level. Finally, a larger sample size may be used to increase behavioral variability.

## 6 Conclusion

Results of this study show that climbers in Metro Manila have a lower tendency to engage in health-seeking behavior. Of the personal factors examined, trust in sources like websites increased the probability of climbers engaging in health-seeking behavior the most. Another factor that boosts the likelihood of seeking healthcare is the preference for a provider who shares an interest in climbing. Moreover, chronic injuries and those located in the lower extremities, specifically the hip/pelvis and knee, were found to augment medical help-seeking behavior. Higher income, educational attainment, and social standing were also associated with an increased likelihood of seeking professional medical assistance.

Given all of this, better monitoring of information on websites is needed as climbers often use this source to augment their professional consultations. One suggestion is to deliver educational healthcare programs which tackle often searched for information online, directly at climbing gyms. Another recommendation is to create educational programs directed at healthcare workers who often encounter climbers. At the end of the day, there is a greater need for healthcare providers to improve the way they approach and deal with the sports climbing community in Metro Manila.

## Data Availability

The raw data supporting the conclusions of this article will be made available by the authors, without undue reservation.
