# Supplementary material for: Factors affecting health-seeking behavior in sports climbers in Metro Manila: a cross-sectional study
Source: Front Sports Act Living. 2025 Feb 24;7:1514827. doi: 10.3389/fspor.2025.1514827 (PMC11891254; doi:10.3389/fspor.2025.1514827)
Supplement: Supplementary file 1 [file Table1.docx]

Supplementary Material

# Supplementary Tables

**Supplementary Table 1.** Operational definitions

|  | **Definition** | **Categories** |
| --- | --- | --- |
| **Social-environmental Factors** | | |
| Age | Current age in years | 16-30 y/o (1)  31-40 y/o (2)  41-50 y/o (3)  51-60 y/o (4) |
| Gender Identity | Current gender identity one relates most to | Male (1)  Female (2)  Trans male/Trans man (3)  Trans female/ Trans woman (4)  Genderqueer/ Gender non-conforming (5)  Other Identity (6) |
| Educational Attainment | Highest educational attainment | No formal schooling (1)  Elementary school graduate (2)  High school graduate (3)  Some college/ no degree (4)  College graduate (5)  Post-graduate degree (6)  Vocational school (7) |
| Occupational Status | Current occupational status | Not employed (1)  Freelance worker/ Self-employed (2)  Part-time employee (3)  Full time employee (4)  Professional (5)  Student (6)  Retired (7) |
| Personal Income | Amount earned through allowances, wages, or professional fees | Estimated average monthly income:  Poor - Less than PHP 9,520.00 (1)  Low Income - PHP 9,520.00 to PHP 19,040.00 (2)  Lower Middle Income - PHP 19,041.00 to PHP 38,080.00 (3)  Middle Income - PHP 38,081.00 to PHP 66,640.00 (4)  Upper Middle Income - PHP 66,641.00 to PHP 114,240.00 (5)  Upper Class - PHP 114,241.00 to PHP 190,040.00 (6)  Rich - At least PHP 190,041.00 (7) |
| **Personal Factors** | | |
| Engagement in Sports Climbing | Duration of engagement in sports climbing | Number of hours in a week spent in sport climbing  1 hour or less (1)  1-3 hours (2)  4-7 hours (3)  8-10 hours (4)  10 or more hours (5) |
|  | Climbing experience | Number of total years spent climbing  Less than 1 year (1)  1-2 years (2)  3-5 years (3)  6-10 years (4)  10 or more years (5) |
| Climbing motivation | Personal goal set during climbing within the past year | Climb harder (1)  Stay at the same level (2)  General fitness (3)  Improved general fitness (4) |
| Climbing Discipline | Climbing style which one practices most frequently | Boulder (1)  Route Climbing (2) |
| Climbing Grade | Highest level of boulder climbing attained | Highest climbing grade attained in boulder  Recreational – Fontainebleau 4 to 5+ (Equivalent to French ≤5+, V0-V2) (1)  Intermediate – Fontainebleau 6A - 6C+ (Equivalent to French 6A - 6C+, V3-V5) (2)  Experienced - Fontainebleau 7A - 7C (Equivalent to French 7A - 7C, V6-V9) (3)  Elite- Fontainebleau 7C+ - 8A+ (Equivalent to French 7C+ - 8A+, V10-V13) (4)  International Elite - Fontainebleau ≥8B (Equivalent to French ≥8B, ≥V13) (5) |
|  | Highest level of route climbing attained | Highest climbing grade attained in route climbing  Recreational - French ≤6b (Equivalent to YDS 5.5-5.10d, Scandinavian 4 - 6+) (1)  Intermediate - French 6b+ - 7a+ (Equivalent to YDS 5.11a-5.12a, Scandinavian 7- - 7+/8-) (2)  Experienced - French 7b - 8b (Equivalent to YDS 5.12b-5.13d, Scandinavian 8- - 9) (3)  Elite - French 8b+ - 8c+ (Equivalent to YDS 5.14a-5.14c, Scandinavian 9/9+ - 9+/10-) (4)  International Elite - French ≥9a (Equivalent to YDS ≥5.14d, Scandinavian ≥10-) (5) |
| Main Training Activities | Physical/ sporting activity which one’s personal time is most spent on | Climbing (1)  Running (2)  Cycling (3)  Strength or weights (4)  Ball-sports (5)  Martial Arts (6)  Gymnastics (7)  Group fitness classes (8)  Skiing or snowboarding (9)  Swimming (10)  Yoga (11)  Others (12) |
| Engagement in Cross-Training Activities | Duration of engagement in cross-training activities | Number of hours in a week spent in cross-training activities:  1 hour or less (1)  1-3 hours (2)  4-7 hours (3)  8-10 hours (4)  10 or more hours (5) |
| Health Seeking Behavior | Preference of provider seen for injuries | Previous provider seen for injuries:  Physician (1)  Physiotherapist (2)  Manual Therapy (3)  Chiropractic (4)  Sports Medicine Specialist (5)  Other Health Professional (6)  Climbing Coach (7)  Coach (8)  Friend (9)  Website (10)  Book (11) Myself (12) |
|  |  | Three most important injury care information source for climbers  Physician (1)  Physiotherapist (2)  Manual Therapy (3)  Chiropractic (4)  Sports Medicine Specialist (5)  Other Health Professional (6)  Climbing Coach (7)  Coach (8)  Friend (9)  Website (10)  Book (11) Myself (12) |
|  |  | Trust in a healthcare provider with climbing experience |
|  |  | Willingness to travel a self an extra hour to see a health care provider with climbing experience |
|  |  | Source who recommended to train at a submaximal level  Health professional (1)  Coach (2) A friend (3)  Internet (4) Myself (5) |
|  | Frequency of use of sources for health care | All the time (1)  Some of the time (2)  Occasionally (3)  Rarely (4)  Never (5) |
|  | Trust in sources for health care | All the time (1)  Some of the time (2)  Occasionally (3)  Rarely (4)  Never (5) |
|  | Trust in climbers with experience of injuries | Strongly agree (1)  Somewhat agree (2)  Neither agree or disagree (3)  Somewhat disagree (4)  Strongly disagree (5) |
|  | Reason for not seeking health care | The injury was not serious enough (1)  Did not have the time (2)  Treated it myself (3)  Do not think health professionals know enough about my climbing-related injuries (4)  Have friends who tried healthcare and it didn't help (5)  A friend helped me (6)  I don't have health insurance (7) |
|  |  | Three most important injury care information sources  Physician (1)  Physiotherapist (2)  Manual Therapy (3)  Chiropractic (4)  Sports Medicine Specialist (5)  Other Health Professional (6)  Climbing Coach (7)  Coach (8)  Friend (9)  Website (10)  Book (11)  Myself (12) |
| **Injury Factors** | | |
| Climbing Injuries | Location of pain | Toe (1)  Foot/ankle (2)  Calf (3)  Knee (4)  Thigh (5)  Hip / Pelvis (6)  Lower back (7)  Abdomen (8)  Chest (9)  Mid/upper back (10)  Neck (11)  Head (12)  Shoulder (13)  Upper arm (14)  Elbow (15)  Forearm (16)  Wrist (17)  Finger (18) |
|  | Duration of pain | Number of days reported in pain  Less than a day (1)  1-3 days (2)  4-7 days (3)  8-28 days (4)  28 or more days (5) |
|  | Location of Injury in the past year | Toe (1)  Foot/ankle (2)  Calf (3)  Knee (4)  Thigh (5)  Hip / Pelvis (6)  Lower back (7)  Abdomen (8)  Chest (9)  Mid/upper back (10)  Neck (11)  Head (12)  Shoulder (13)  Upper arm (14)  Elbow (15)  Forearm (16)  Wrist (17)  Finger (18) |
|  | Duration of Injury | Number of days injured:  Less than a day (1)  1-3 days (2)  4-7 days (3)  8-28 days (4)  28 or more days (5) |
|  | Severity of Injury | Ability to return to play  Yes (1)  No (2)  Trained at a lower level (submaximal training) (3) |
|  |  | Duration of Return to Play (Stopped training)  Less than a week (1)  1-2 weeks (2)  3-4 weeks (3)  1-3 months (4)  4-6 months (5)  More than 6 months (6) |
|  |  | Duration of Submaximal training (Trained at reduced level)  Less than a week (1)  1-2 weeks (2)  3-4 weeks (3)  1-3 months (4)  4-6 months (5)  More than 6 months (6) |
|  |  | Who diagnosed the injury  Physician (1)  PT (2)  Non-PT manual therapist (3)  Chiropractor (4)  Sports medicine specialist (Non-MD) (5)  Other health professional (6)  Climbing coach (7)  Other non-climbing coach (8)  Friend (9)  Website (10)  Book (11)  Myself (Self-diagnosed) (12) |

**Supplementary Table 2.** Socio-Demographic Characteristics of Sports Climbers in Metro Manila

|  | **All** | **With Hand Injuries** | **Without Hand Injuries** | **p value** |
| --- | --- | --- | --- | --- |
| **n** | **125** | **94** | **31** |  |
| **Age** |  |  |  |  |
| 16-30 | 91 (72.8) | 71 (75.5) | 20 (64.5) | 0.4545 |
| 31-40 | 26 (20.8) | 18 (19.1) | 8 (25.8) |  |
| 41-50 | 8 (6.4) | 5 (5.3) | 3 (9.7) |  |
| **Gender Identity** |  |  |  |  |
| Male | 62 (49.6) | 50 (53.2) | 12 (38.7) | 0.1327 |
| Female | 59 (47.2) | 40 (42.6) | 19 (61.3) |  |
| Genderqueer/ Gender non-conforming | 4 (3.2) | 4 (4.3) | 0 (0.0) |  |
| **Highest Educational Attainment** |  |  |  |  |
| No formal schooling | 1 (0.8) |  |  |  |
| Elementary school graduate | 3 (2.4) | 3 (3.2) | 0 (0.0) | 0.3529 |
| High school graduate | 10 (8) | 9 (9.6) | 1 (3.2) |  |
| Some college/ no degree | 9 (7.2) | 7 (7.4) | 2 (6.5) |  |
| College graduate | 84 (67.2) | 60 (63.8) | 24 (77.4) |  |
| Post-graduate degree | 17 (13.6) | 14 (14.9) | 3 (9.7) |  |
| Vocational school | 1 (0.8) | 1 (1.1) | 0 (0.0) |  |
| **Occupational Status** |  |  |  |  |
| Not employed | 3 (2.4) | 1 (1.1) | 2 (6.5) | 0.2844 |
| Freelance worker/ Self-employed | 22 (17.6) | 15 (16.0) | 7 (22.6) |  |
| Part-time employee | 3 (2.4) | 2 (2.1) | 1 (3.2) |  |
| Full time employee | 68 (54.4) | 54 (57.4) | 14 (45.2) |  |
| Professional | 10 (8) | 6 (6.4) | 4 (12.9) |  |
| Student | 19 (15.2) | 16 (17.0) | 3 (9.7) |  |
| **Estimated average monthly income:** |  |  |  |  |
| Less than PHP 9,520.00 | 15 (12) | 10 (10.6) | 1 (3.2) | 0.0552 |
| PHP 9,520.00 to PHP 19,040.00 | 11 (8.8) | 11 (11.7) | 0 (0.0) |  |
| PHP 19,041.00 to PHP 38,080.00 | 28 (22.4) | 22 (23.4) | 6 (19.4) |  |
| PHP 38,081.00 to PHP 66,640.00 | 27 (21.6) | 15 (16.0) | 12 (38.7) |  |
| PHP 66,641.00 to PHP 114,240.00 | 24 (19.2) | 18 (19.1) | 6 (19.4) |  |
| PHP 114,241.00 to PHP 190,040.00 | 10 (8) | 9 (9.6) | 1 (3.2) |  |
| At least PHP 190,041.00 | 10 (8) | 9 (9.6) | 1 (3.2) |  |

**Supplementary Table 3.** Socio-Demographic Factors associated with Attitudes Towards Medical Help-seeking Scale in Sports Climbers in Metro Manila

|  | **All** | **Action-intention Subscale Score range** | | | |
| --- | --- | --- | --- | --- | --- |
|  |  | **0-12** | **13-24** | **25-36** | **p value** |
| n |  | **3** | **65** | **57** |  |
| ***Socio-Demographic*** |  |  |  |  |  |
| **Age in Years, n (%)** |  |  |  |  |  |
| 16-30 | 91 (72.8) | 3 (100) | 53 (81.5) | 35 (61.4) | 0.1168 |
| 31-40 | 26 (20.8) | 0 (0.0) | 9 (13.8) | 17 (29.8) |  |
| 41-50 | 8 (6.4) | 0 (0.0) | 3 (4.6) | 5 (8.8) |  |
| **Gender Identity, n (%)** |  |  |  |  |  |
| Male | 62 (49.6) | 1 (33.3) | 35 (53.8) | 26 (45.6) | 0.646 |
| Female | 59 (47.2) | 2 (66.7) | 27 (41.5) | 30 (52.6) |  |
| Genderqueer/ Gender non-conforming | 4 (3.2) | 0 (0.0) | 3 (4.6) | 1 (1.8) |  |
| **Highest Educational Attainment, n (%)** |  |  |  |  |  |
| No formal schooling | 1 (0.8) | 0 (0.0) | 0 (0.0) | 1 (1.8) | 0.8342 |
| Elementary school graduate | 3 (2.4) | 0 (0.0) | 1 (1.5) | 2 (3.5) |  |
| High school graduate | 10 (8) | 1 (33.3) | 6 (9.2) | 3 (5.3) |  |
| Some college/ no degree | 9 (7.2) | 0 (0.0) | 6 (9.2) | 3 (5.3) |  |
| College graduate | 84 (67.2) | 2 (66.7) | 43 (66.2) | 39 (68.4) |  |
| Post-graduate degree | 17 (13.6) | 0 (0.0) | 8 (12.3) | 9 (15.8) |  |
| Vocational school | 1 (0.8) | 0 (0.0) | 1 (1.5) | 0 (0.0) |  |
| **Occupational Status, n (%)** |  |  |  |  |  |
| Not employed | 3 (2.4) | 0 (0.0) | 3 (4.6) | 0 (0.0) | 0.7574 |
| Freelance worker/ Self-employed | 22 (17.6) | 0 (0.0) | 14 (21.5) | 8 (14.0) |  |
| Part-time employee | 3 (2.4) | 0 (0.0) | 2 (3.1) | 1 (1.8) |  |
| Full time employee | 68 (54.4) | 2 (66.7) | 33 (50.8) | 33 (57.9) |  |
| Professional | 10 (8) | 0 (0.0) | 4 (6.2) | 6 (10.5) |  |
| Student | 19 (15.2) | 1 (33.3) | 9 (13.8) | 9 (15.8) |  |
| **Estimated average monthly income, n (%)** |  |  |  |  |  |
| Less than PHP 9,520.00 | 15 (12) | 1 (33.3) | 11 (16.9) | 3 (5.3) | 0.3193 |
| PHP 9,520.00 to PHP 19,040.00 | 11 (8.8) | 0 (0.0) | 7 (10.8) | 4 (7.0) |  |
| PHP 19,041.00 to PHP 38,080.00 | 28 (22.4) | 0 (0.0) | 16 (24.6) | 12 (21.1) |  |
| PHP 38,081.00 to PHP 66,640.00 | 27 (21.6) | 2 (66.7) | 13 (20.0) | 12 (21.1) |  |
| PHP 66,641.00 to PHP 114,240.00 | 24 (19.2) | 0 (0.0) | 11 (16.9) | 13 (22.8) |  |
| PHP 114,241.00 to PHP 190,040.00 | 10 (8) | 0 (0.0) | 4 (6.2) | 6 (10.5) |  |
| At least PHP 190,041.00 | 10 (8) | 0 (0.0) | 3 (4.6) | 7 (12.3) |  |

**Supplementary Table 4.** Personal Factors associated with Attitudes Towards Medical Help-seeking Scale in Sports Climbers in Metro Manila

| ***Personal factors*** | **All** | **Action-intention Subscale Score range** | | | |
| --- | --- | --- | --- | --- | --- |
| **Main Training Activities, n (%)** |  | **0-12** | **13-24** | **25-36** | **p value** |
| Climbing | 119 (95.2) | 3 (100) | 62 (95.4) | 54 (94.7) | 0.9126 |
| Running | 34 (27.2) | 0 (0.0) | 18 (27.7) | 16 (28.1) | 0.5625 |
| Cycling | 18 (14.4) | 1 (33.3) | 10 (15.4) | 7 (12.3) | 0.568 |
| Weight training | 67 (53.6) | 2 (66.7) | 32 (49.2) | 33 (57.9) | 0.569 |
| Ball-sports | 13 (10.4) | 0 (0.0) | 7 (10.8) | 6 (10.5) | 0.8358 |
| Martial arts | 4 (3.2) | 0 (0.0) | 3 (4.6) | 1 (1.8) | 0.6363 |
| Gymnastics | 1 (0.8) | 0 (0.0) | 0 (0.0) | 1 (1.8) | 0.5481 |
| Group fitness classes | 5 (4) | 0 (0.0) | 3 (4.6) | 2 (3.5) | 0.8936 |
| Skiing/ snowboarding | 1 (0.8) | 0 (0.0) | 0 (0.0) | 1 (1.8) | 0.5481 |
| Swimming | 5 (4) | 0 (0.0) | 2 (3.1) | 3 (5.3) | 0.7764 |
| Yoga | 17 (13.6) | 2 (66.7) | 8 (12.3) | 7 (12.3) | **0.0251** |
| Others | 17 (13.6) | 1 (33.3) | 6 (9.2) | 10 (17.5) | 0.246 |
| **Main climbing discipline, n (%)** |  |  |  |  |  |
| Boulder | 78 (62.4) | 3 (100) | 43 (66.2) | 32 (56.1) | 0.207 |
| Route Climber | 47 (37.6) | 0 (0.0) | 22 (33.8) | 25 (43.9) |  |
| **Highest level of bouldering climbing performance, n (%)** |  |  |  |  |  |
| Recreational: Fontainebleau 4 to 5+ (1) | 15 (19.2) | 1 (33.3) | 10 (23.3) | 4 (12.5) | 0.7795 |
| Intermediate: Fontainebleau 6A to 6C+ (2) | 38 (48.7) | 1 (33.3) | 18 (41.9) | 19 (59.4) |  |
| Experienced: Fontainebleau 7A to 7C (3) | 23 (29.5) | 1 (33.3) | 14 (32.6) | 8 (25.0) |  |
| Elite: Fontainebleau 7C+ to 8A+ (4) | 2 (2.6) | 0 (0.0) | 1 (2.3) | 1 (3.1) |  |
| International Elite: Fontainebleau ≥8B (5) | 0 (0) | 0 (0.0) | 0 (0.0) | 0 (0.0) |  |
| **Highest level of route climbing performance, n (%)** |  |  |  |  |  |
| Recreational: French 4 to 6b (1) | 20 (42.6) | 0 (0.0) | 11 (50.0) | 9 (36.0) | 0.4425 |
| Intermediate: French 6b+ - 7a+ (2) | 26 (55.3) | 0 (0.0) | 11 (50.0) | 15 (60.0) |  |
| Experienced: French 7b - 8b (3) | 1 (2.1) | 0 (0.0) | 0 (0.0) | 1 (4.0) |  |
| Elite: French 8b+ - 8c+ (4) | 0 (0) | 0 (0.0) | 0 (0.0) | 0 (0.0) |  |
| International Elite: French ≥9a (5) | 0 (0) | 0 (0.0) | 0 (0.0) | 0 (0.0) |  |
| **Hours per week of climbing in total, n (%)** |  |  |  |  |  |
| 1 hr or less (1) | 7 (5.6) | 0 (0.0) | 5 (7.7) | 2 (3.5) | 0.9449 |
| 1-3 hrs (2) | 24 (19.2) | 1 (33.3) | 11 (16.9) | 12 (21.1) |  |
| 4-7 hrs (3) | 41 (32.8) | 1 (33.3) | 22 (33.8) | 18 (31.6) |  |
| 8-10 hrs (4) | 30 (24) | 1 (33.3) | 14 (21.5) | 15 (26.3) |  |
| 10 or more hrs (5) | 23 (18.4) | 0 (0.0) | 13 (20.0) | 10 (17.5) |  |
| **Hours a week spent training non-climbing in total, n (%)** |  |  |  |  |  |
| 1 hr or less (1) | 6 (4.8) | 0 (0.0) | 4 (6.2) | 2 (3.5) | 0.6764 |
| 1-3 hrs (2) | 41 (32.8) | 1 (33.3) | 21 (32.3) | 19 (33.3) |  |
| 4-7 hrs (3) | 51 (40.8) | 2 (66.7) | 28 (43.1) | 21 (36.8) |  |
| 8-10 hrs (4) | 16 (12.8) | 0 (0.0) | 5 (7.7) | 11 (19.3) |  |
| 10 or more hrs (5) | 11 (8.8) | 0 (0.0) | 7 (10.8) | 4 (7.0) |  |
| **Total years spent climbing, n (%)** |  |  |  |  |  |
| Less than 1 yr (1) | 48 (38.4) | 2 (66.7) | 26 (40.0) | 20 (35.1) | 0.5657 |
| 1-2 yrs (2) | 44 (35.2) | 1 (33.3) | 25 (38.5) | 18 (31.6) |  |
| 3-5 yrs (3) | 9 (7.2) | 0 (0.0) | 6 (9.2) | 3 (5.3) |  |
| 6-10 yrs (4) | 16 (12.8) | 0 (0.0) | 5 (7.7) | 11 (19.3) |  |
| 10 or more yrs (5) | 8 (6.4) | 0 (0.0) | 3 (4.6) | 5 (8.8) |  |
| **Frequency of use of sources for health care: Physician, n (%)** |  |  |  |  |  |
| *Mean ± SD* | 3.79 ± 1.27 | 4.67 ± 0.58 | 3.98 ± 1.12 | 3.53 ± 1.40 | 0.1836 |
| All the time (1) | 7 (5.6) | 0 (0.0) | 0 (0.0) | 7 (12.3) |  |
| Some of the time (2) | 18 (14.4) | 0 (0.0) | 10 (15.4) | 8 (14.0) |  |
| Occasionally (3) | 20 (16) | 0 (0.0) | 11 (16.9) | 9 (15.8) |  |
| Rarely (4) | 29 (23.2) | 1 (33.3) | 14 (21.5) | 14 (24.6) |  |
| Never (5) | 51 (40.8) | 2 (66.7) | 30 (46.2) | 19 (33.3) |  |
| **Frequency of use of sources for health care: PT, n (%)** |  |  |  |  |  |
| *Mean ± SD* | 3.86 ± 1.23 | 4.67 ± 0.58 | 3.98 ± 1.10 | 3.68 ± 1.38 | 0.5875 |
| All the time (1) | 7 (5.6) | 0 (0.0) | 1 (1.5) | 6 (10.5) |  |
| Some of the time (2) | 14 (11.2) | 0 (0.0) | 7 (10.8) | 7 (12.3) |  |
| Occasionally (3) | 20 (16) | 0 (0.0) | 12 (18.5) | 8 (14.0) |  |
| Rarely (4) | 32 (25.6) | 1 (33.3) | 17 (26.2) | 14 (24.6) |  |
| Never (5) | 52 (41.6) | 2 (66.7) | 28 (43.1) | 22 (38.6) |  |
| **Frequency of use of sources for health care: Non-PT manual therapist, n (%)** |  |  |  |  |  |
| *Mean ± SD* | 4.46 ± 0.96 | 5.00 ± 0.00 | 4.52 ± 0.87 | 4.35 ± 1.08 | 0.905 |
| All the time (1) | 1 (0.8) | 0 (0.0) | 0 (0.0) | 1 (1.8) |  |
| Some of the time (2) | 7 (5.6) | 0 (0.0) | 3 (4.6) | 4 (7.0) |  |
| Occasionally (3) | 15 (12) | 0 (0.0) | 7 (10.8) | 8 (14.0) |  |
| Rarely (4) | 13 (10.4) | 0 (0.0) | 8 (12.3) | 5 (8.8) |  |
| Never (5) | 89 (71.2) | 3 (100) | 47 (72.3) | 39 (68.4) |  |
| **Frequency of use of sources for health care: Sports medicine specialist (Non-MD), n (%)** |  |  |  |  |  |
| *Mean ± SD* | 3.82 ± 1.30 | 4.33 ± 1.15 | 4.03 ± 1.22 | 3.56 ± 1.35 | 0.6031 |
| All the time (1) | 11 (8.8) | 0 (0.0) | 4 (6.2) | 7 (12.3) |  |
| Some of the time (2) | 8 (6.4) | 0 (0.0) | 4 (6.2) | 4 (7.0) |  |
| Occasionally (3) | 27 (21.6) | 1 (33.3) | 11 (16.9) | 15 (26.3) |  |
| Rarely (4) | 25 (20) | 0 (0.0) | 13 (20.0) | 12 (21.1) |  |
| Never (5) | 54 (43.2) | 2 (66.7) | 33 (50.8) | 19 (33.3) |  |
| **Frequency of use of sources for health care: Other health professional, n (%)** |  |  |  |  |  |
| *Mean ± SD* | 4.22 ± 1.01 | 4.33 ± 0.58 | 4.23 ± 1.04 | 4.21 ± 1.01 | 0.614 |
| All the time (1) | 1 (0.8) | 0 (0.0) | 1 (1.5) | 0 (0.0) |  |
| Some of the time (2) | 8 (6.4) | 0 (0.0) | 4 (6.2) | 4 (7.0) |  |
| Occasionally (3) | 23 (18.4) | 0 (0.0) | 11 (16.9) | 12 (21.1) |  |
| Rarely (4) | 23 (18.4) | 2 (66.7) | 12 (18.5) | 9 (15.8) |  |
| Never (5) | 70 (56) | 1 (33.3) | 37 (56.9) | 32 (56.1) |  |
| **Frequency of use of sources for health care: Climbing coach, n (%)** |  |  |  |  |  |
| *Mean ± SD* | 3.98 ± 1.22 | 4.67 ± 0.58 | 4.08 ± 1.08 | 3.82 ± 1.38 | 0.2203 |
| All the time (1) | 5 (4) | 0 (0.0) | 0 (0.0) | 5 (8.8) |  |
| Some of the time (2) | 12 (9.6) | 0 (0.0) | 7 (10.8) | 5 (8.8) |  |
| Occasionally (3) | 28 (22.4) | 0 (0.0) | 14 (21.5) | 14 (24.6) |  |
| Rarely (4) | 16 (12.8) | 1 (33.3) | 11 (16.9) | 4 (7.0) |  |
| Never (5) | 64 (51.2) | 2 (66.7) | 33 (50.8) | 29 (50.9) |  |
| **Frequency of use of sources for health care: Non-Climbing coach, n (%)** |  |  |  |  |  |
| *Mean ± SD* | 4.18 ± 1.10 | 4.67 ± 0.58 | 4.29 ± 0.96 | 4.02 ± 1.25 | 0.7124 |
| All the time (1) | 3 (2.4) | 0 (0.0) | 0 (0.0) | 3 (5.3) |  |
| Some of the time (2) | 9 (7.2) | 0 (0.0) | 4 (6.2) | 5 (8.8) |  |
| Occasionally (3) | 21 (16.8) | 0 (0.0) | 11 (16.9) | 10 (17.5) |  |
| Rarely (4) | 22 (17.6) | 1 (33.3) | 12 (18.5) | 9 (15.8) |  |
| Never (5) | 70 (56) | 2 (66.7) | 38 (58.5) | 30 (52.6) |  |
| **Frequency of use of sources for health care: Friend, n (%)** |  |  |  |  |  |
| *Mean ± SD* | 3.13 ± 1.28 | 2.00 ± 1.00 | 3.05 ± 1.23 | 3.28 ± 1.32 | 0.6564 |
| All the time (1) | 16 (12.8) | 1 (33.3) | 8 (12.3) | 7 (12.3) |  |
| Some of the time (2) | 23 (18.4) | 1 (33.3) | 14 (21.5) | 8 (14.0) |  |
| Occasionally (3) | 38 (30.4) | 1 (33.3) | 19 (29.2) | 18 (31.6) |  |
| Rarely (4) | 25 (20) | 0 (0.0) | 15 (23.1) | 10 (17.5) |  |
| Never (5) | 23 (18.4) | 0 (0.0) | 9 (13.8) | 14 (24.6) |  |
| **Frequency of use of sources for health care: Website, n (%)** |  |  |  |  |  |
| *Mean ± SD* | 2.85 ± 1.35 | 1.67 ± 1.15 | 2.75 ± 1.25 | 3.02 ± 1.45 | 0.3181 |
| All the time (1) | 22 (17.6) | 2 (66.7) | 10 (15.4) | 10 (17.5) |  |
| Some of the time (2) | 35 (28) | 0 (0.0) | 21 (32.3) | 14 (24.6) |  |
| Occasionally (3) | 31 (24.8) | 1 (33.3) | 18 (27.7) | 12 (21.1) |  |
| Rarely (4) | 14 (11.2) | 0 (0.0) | 7 (10.8) | 7 (12.3) |  |
| Never (5) | 23 (18.4) | 0 (0.0) | 9 (13.8) | 14 (24.6) |  |
| **Frequency of use of sources for health care: Books, n (%)** |  |  |  |  |  |
| *Mean ± SD* | 4.02 ± 1.24 | 3.33 ± 2.08 | 4.15 ± 1.14 | 3.91 ± 1.31 | 0.2732 |
| All the time (1) | 6 (4.8) | 1 (33.3) | 2 (3.1) | 3 (5.3) |  |
| Some of the time (2) | 11 (8.8) | 0 (0.0) | 4 (6.2) | 7 (12.3) |  |
| Occasionally (3) | 25 (20) | 0 (0.0) | 13 (20.0) | 12 (21.1) |  |
| Rarely (4) | 15 (12) | 1 (33.3) | 9 (13.8) | 5 (8.8) |  |
| Never (5) | 68 (54.4) | 1 (33.3) | 37 (56.9) | 30 (52.6) |  |
| **Frequency of use of sources for health care: Myself, n (%)** |  |  |  |  |  |
| *Mean ± SD* | 2.82 ± 1.36 | 3.67 ± 1.15 | 2.72 ± 1.29 | 2.88 ± 1.45 | 0.4779 |
| All the time (1) | 25 (20) | 0 (0.0) | 13 (20.0) | 12 (21.1) |  |
| Some of the time (2) | 32 (25.6) | 0 (0.0) | 18 (27.7) | 14 (24.6) |  |
| Occasionally (3) | 31 (24.8) | 2 (66.7) | 16 (24.6) | 13 (22.8) |  |
| Rarely (4) | 15 (12) | 0 (0.0) | 10 (15.4) | 5 (8.8) |  |
| Never (5) | 22 (17.6) | 1 (33.3) | 8 (12.3) | 13 (22.8) |  |
| **Trust in sources for health care: Physician, n (%)** |  |  |  |  |  |
| *Mean ± SD* | 2.16 ± 1.10 | 2.00 ± 1.00 | 2.09 ± 1.07 | 2.25 ± 1.14 | 0.9938 |
| All the time (1) | 44 (35.2) | 1 (33.3) | 24 (36.9) | 19 (33.3) |  |
| Some of the time (2) | 35 (28) | 1 (33.3) | 19 (29.2) | 15 (26.3) |  |
| Occasionally (3) | 32 (25.6) | 1 (33.3) | 16 (24.6) | 15 (26.3) |  |
| Rarely (4) | 10 (8) | 0 (0.0) | 4 (6.2) | 6 (10.5) |  |
| Never (5) | 4 (3.2) | 0 (0.0) | 2 (3.1) | 2 (3.5) |  |
| **Trust in sources for health care: PT, n (%)** |  |  |  |  |  |
| *Mean ± SD* | 1.76 ± 1.06 | 1.33 ± 0.58 | 1.72 ± 0.99 | 1.82 ± 1.15 | 0.9767 |
| All the time (1) | 69 (55.2) | 2 (66.7) | 36 (55.4) | 31 (54.4) |  |
| Some of the time (2) | 32 (25.6) | 1 (33.3) | 17 (26.2) | 14 (24.6) |  |
| Occasionally (3) | 13 (10.4) | 0 (0.0) | 7 (10.8) | 6 (10.5) |  |
| Rarely (4) | 7 (5.6) | 0 (0.0) | 4 (6.2) | 3 (5.3) |  |
| Never (5) | 4 (3.2) | 0 (0.0) | 1 (1.5) | 3 (5.3) |  |
| **Trust in sources for health care: Non-PT Manual Therapist, n (%)** |  |  |  |  |  |
| *Mean ± SD* | 3.23 ± 1.37 | 4.00 ± 1.00 | 3.34 ± 1.30 | 3.07 ± 1.45 | 0.598 |
| All the time (1) | 16 (12.8) | 0 (0.0) | 7 (10.8) | 9 (15.8) |  |
| Some of the time (2) | 25 (20) | 0 (0.0) | 10 (15.4) | 15 (26.3) |  |
| Occasionally (3) | 30 (24) | 1 (33.3) | 18 (27.7) | 11 (19.3) |  |
| Rarely (4) | 22 (17.6) | 1 (33.3) | 14 (21.5) | 7 (12.3) |  |
| Never (5) | 32 (25.6) | 1 (33.3) | 16 (24.6) | 15 (26.3) |  |
| **Trust in sources for health care: Sports medicine specialist (Non-MD), n (%)** |  |  |  |  |  |
| *Mean ± SD* | 1.68 ± 1.08 | 1.00 ± 0.00 | 1.83 ± 1.14 | 1.54 ± 1.02 | 0.5994 |
| All the time (1) | 77 (61.6) | 3 (100) | 34 (52.3) | 40 (70.2) |  |
| Some of the time (2) | 27 (21.6) | 0 (0.0) | 18 (27.7) | 9 (15.8) |  |
| Occasionally (3) | 11 (8.8) | 0 (0.0) | 7 (10.8) | 4 (7.0) |  |
| Rarely (4) | 4 (3.2) | 0 (0.0) | 2 (3.1) | 2 (3.5) |  |
| Never (5) | 6 (4.8) | 0 (0.0) | 4 (6.2) | 2 (3.5) |  |
| **Trust in sources for health care: Other Health Professionals , n (%)** |  |  |  |  |  |
| *Mean ± SD* | 2.65 ± 1.17 | 2.33 ± 1.15 | 2.69 ± 1.14 | 2.61 ± 1.22 | 0.8658 |
| All the time (1) | 22 (17.6) | 1 (33.3) | 10 (15.4) | 11 (19.3) |  |
| Some of the time (2) | 37 (29.6) | 0 (0.0) | 19 (29.2) | 18 (31.6) |  |
| Occasionally (3) | 41 (32.8) | 2 (66.7) | 23 (35.4) | 16 (28.1) |  |
| Rarely (4) | 13 (10.4) | 0 (0.0) | 7 (10.8) | 6 (10.5) |  |
| Never (5) | 12 (9.6) | 0 (0.0) | 6 (9.2) | 6 (10.5) |  |
| **Trust in sources for health care: Climbing coach, n (%)** |  |  |  |  |  |
| *Mean ± SD* | 2.36 ± 1.03 | 1.67 ± 0.58 | 2.35 ± 0.96 | 2.40 ± 1.12 | 0.1836 |
| All the time (1) | 7 (5.6) | 0 (0.0) | 0 (0.0) | 7 (12.3) |  |
| Some of the time (2) | 18 (14.4) | 0 (0.0) | 10 (15.4) | 8 (14.0) |  |
| Occasionally (3) | 20 (16) | 0 (0.0) | 11 (16.9) | 9 (15.8) |  |
| Rarely (4) | 29 (23.2) | 1 (33.3) | 14 (21.5) | 14 (24.6) |  |
| Never (5) | 51 (40.8) | 2 (66.7) | 30 (46.2) | 19 (33.3) |  |
| **Trust in sources for health care: Non-climbing coach, n (%)** |  |  |  |  |  |
| *Mean ± SD* | 2.92 ± 1.26 | 4.00 ± 1.00 | 2.88 ± 1.24 | 2.91 ± 1.29 | 0.9042 |
| All the time (1) | 16 (12.8) | 0 (0.0) | 8 (12.3) | 8 (14.0) |  |
| Some of the time (2) | 35 (28) | 0 (0.0) | 20 (30.8) | 15 (26.3) |  |
| Occasionally (3) | 38 (30.4) | 1 (33.3) | 19 (29.2) | 18 (31.6) |  |
| Rarely (4) | 15 (12) | 1 (33.3) | 8 (12.3) | 6 (10.5) |  |
| Never (5) | 21 (16.8) | 1 (33.3) | 10 (15.4) | 10 (17.5) |  |
| **Trust in sources for health care: Friend, n (%)** |  |  |  |  |  |
| *Mean ± SD* | 3.14 ± 1.00 | 2.67 ± 1.15 | 3.02 ± 0.98 | 3.32 ± 1.00 | 0.0986 |
| All the time (1) | 6 (4.8) | 0 (0.0) | 4 (6.2) | 2 (3.5) |  |
| Some of the time (2) | 24 (19.2) | 2 (66.7) | 15 (23.1) | 7 (12.3) |  |
| Occasionally (3) | 53 (42.4) | 0 (0.0) | 25 (38.5) | 28 (49.1) |  |
| Rarely (4) | 30 (24) | 1 (33.3) | 18 (27.7) | 11 (19.3) |  |
| Never (5) | 12 (9.6) | 0 (0.0) | 3 (4.6) | 9 (15.8) |  |
| **Trust in sources for health care: Website, n (%)** |  |  |  |  |  |
| *Mean ± SD* | 2.99 ± 1.00 | 2.33 ± 1.53 | 2.82 ± 0.86 | 3.23 ± 1.09 | **0.0378** |
| All the time (1) | 7 (5.6) | 1 (33.3) | 4 (6.2) | 2 (3.5) |  |
| Some of the time (2) | 31 (24.8) | 1 (33.3) | 18 (27.7) | 12 (21.1) |  |
| Occasionally (3) | 54 (43.2) | 0 (0.0) | 30 (46.2) | 24 (42.1) |  |
| Rarely (4) | 22 (17.6) | 1 (33.3) | 12 (18.5) | 9 (15.8) |  |
| Never (5) | 11 (8.8) | 0 (0.0) | 1 (1.5) | 10 (17.5) |  |
| **Trust in sources for health care: Book, n (%)** |  |  |  |  |  |
| *Mean ± SD* | 3.35 ± 1.23 | 3.33 ± 2.08 | 3.18 ± 1.22 | 3.54 ± 1.20 | 0.3045 |
| All the time (1) | 9 (7.2) | 1 (33.3) | 6 (9.2) | 2 (3.5) |  |
| Some of the time (2) | 22 (17.6) | 0 (0.0) | 13 (20.0) | 9 (15.8) |  |
| Occasionally (3) | 41 (32.8) | 0 (0.0) | 21 (32.3) | 20 (35.1) |  |
| Rarely (4) | 22 (17.6) | 1 (33.3) | 13 (20.0) | 8 (14.0) |  |
| Never (5) | 31 (24.8) | 1 (33.3) | 12 (18.5) | 18 (31.6) |  |
| **Trust in sources for health care: Myself, n (%)** |  |  |  |  |  |
| *Mean ± SD* | 3.34 ± 1.25 | 4.67 ± 0.58 | 3.31 ± 1.16 | 3.32 ± 1.35 | 0.1846 |
| All the time (1) | 10 (8) | 0 (0.0) | 3 (4.6) | 7 (12.3) |  |
| Some of the time (2) | 23 (18.4) | 0 (0.0) | 15 (23.1) | 8 (14.0) |  |
| Occasionally (3) | 36 (28.8) | 0 (0.0) | 18 (27.7) | 18 (31.6) |  |
| Rarely (4) | 26 (20.8) | 1 (33.3) | 17 (26.2) | 8 (14.0) |  |
| Never (5) | 30 (24) | 2 (66.7) | 12 (18.5) | 16 (28.1) |  |
| **I trust climbers with experience of a similar pain/injury more than health providers, n (%)** |  |  |  |  |  |
| *Mean ± SD* | 3.1 ± 1.0 | 2.67 ± 0.58 | 3.00 ± 0.88 | 3.26 ± 1.08 | 0.4374 |
| Strongly agree (1) | 5 (4) | 0 (0.0) | 2 (3.1) | 3 (5.3) |  |
| Somewhat agree (2) | 28 (22.4) | 1 (33.3) | 17 (26.2) | 10 (17.5) |  |
| Neither agree nor disagree (3) | 50 (40) | 2 (66.7) | 27 (41.5) | 21 (36.8) |  |
| Somewhat disagree (4) | 32 (25.6) | 0 (0.0) | 17 (26.2) | 15 (26.3) |  |
| Strongly disagree (5) | 10 (8) | 0 (0.0) | 2 (3.1) | 8 (14.0) |  |
| **If possible, I would rather travel for an extra hour to see a healthcare provider that is a climber, n (%)** | |  |  |  |  |
| *Mean ± SD* | 2.0 ± 1.2 | 2.33 ± 2.31 | 2.09 ± 1.13 | 1.95 ± 1.17 | 0.092 |
| Strongly agree (1) | 54 (43.2) | 2 (66.7) | 26 (40.0) | 26 (45.6) |  |
| Somewhat agree (2) | 36 (28.8) | 0 (0.0) | 18 (27.7) | 18 (31.6) |  |
| Neither agree nor disagree (3) | 18 (14.4) | 0 (0.0) | 11 (16.9) | 7 (12.3) |  |
| Somewhat disagree (4) | 11 (8.8) | 0 (0.0) | 9 (13.8) | 2 (3.5) |  |
| Strongly disagree (5) | 6 (4.8) | 1 (33.3) | 1 (1.5) | 4 (7.0) |  |
| **Why did you choose not to seek professional health care** | |  |  |  |  |
| I Don't have health insurance | 1 (6.2) | 0 (0.0) | 1 (12.5) | 0 (0.0) | 0.3679 |
| I treated it myself | 1 (6.2) | 0 (0.0) | 0 (0.0) | 1 (12.5) |  |
| The injury was not serious enough | 14 (87.5) | 0 (0.0) | 7 (87.5) | 7 (87.5) |  |

**Supplementary Table 5.** Injury Factors associated with Attitudes Towards Medical Help-seeking Scale in Sports Climbers in Metro Manila

| ***Injury factors*** | **All** | **Action-intention Subscale Score range** | | | |
| --- | --- | --- | --- | --- | --- |
| **Presence of climbing related injury (within the last year), n (%)** |  | **0-12** | **13-24** | **25-36** | **p value** |
| Yes (1) | 94 (75.2) | 2 (66.7) | 50 (76.9) | 42 (73.7) | 0.8647 |
| No (2) | 31 (24.8) | 1 (33.3) | 15 (23.1) | 15 (26.3) |  |
| **Location of pain, n (%)** |  |  |  |  |  |
| Toe (1) | 12 (9.6) | 0 (0.0) | 8 (12.3) | 4 (7.0) | 0.5206 |
| Foot/ankle (2) | 30 (24) | 1 (33.3) | 16 (24.6) | 13 (22.8) | 0.9043 |
| Calf (3) | 5 (4) | 0 (0.0) | 5 (7.7) | 0 (0.0) | 0.0904 |
| Knee (4) | 27 (21.6) | 2 (66.7) | 17 (26.2) | 8 (14.0) | **0.0424** |
| Thigh (5) | 10 (8) | 0 (0.0) | 7 (10.8) | 3 (5.3) | 0.4681 |
| Hip/ Pelvis (6) | 14 (11.2) | 2 (66.7) | 7 (10.8) | 5 (8.8) | **0.0081** |
| Lower back (7) | 15 (12) | 1 (33.3) | 9 (13.8) | 5 (8.8) | 0.3561 |
| Abdomen (8) | 1 (0.8) | 0 (0.0) | 1 (1.5) | 0 (0.0) | 0.628 |
| Chest (9) | 3 (2.4) | 0 (0.0) | 1 (1.5) | 2 (3.5) | 0.7487 |
| Mid/ upper back (10) | 25 (20) | 0 (0.0) | 15 (23.1) | 10 (17.5) | 0.5093 |
| Neck (11) | 11 (8.8) | 1 (33.3) | 5 (7.7) | 5 (8.8) | 0.3089 |
| Head (12) | 0 (0) | 0 (0.0) | 0 (0.0) | 0 (0.0) | - |
| Shoulder (13) | 43 (34.4) | 2 (66.7) | 20 (30.8) | 21 (36.8) | 0.384 |
| Forearm (14) | 31 (24.8) | 1 (33.3) | 20 (30.8) | 10 (17.5) | 0.2267 |
| Wrist (15) | 48 (38.4) | 2 (66.7) | 26 (40.0) | 20 (35.1) | 0.5096 |
| Finger (16) | 58 (46.4) | 2 (66.7) | 29 (44.6) | 27 (47.4) | 0.7407 |
| **Location of injury, n (%)** |  |  |  |  |  |
| Toe (1) | 4 (3.2) | 0 (0.0) | 3 (4.6) | 1 (1.8) | 0.6363 |
| Foot/ankle (2) | 5 (4) | 0 (0.0) | 3 (4.6) | 2 (3.5) | 0.8936 |
| Calf (3) | 1 (0.8) | 0 (0.0) | 1 (1.5) | 0 (0.0) | 0.628 |
| Knee (4) | 4 (3.2) | 0 (0.0) | 2 (3.1) | 2 (3.5) | 0.9418 |
| Thigh (5) | 0 (0) | 0 (0.0) | 0 (0.0) | 0 (0.0) | - |
| Hip/ Pelvis (6) | 5 (4) | 0 (0.0) | 1 (1.5) | 4 (7.0) | 0.2862 |
| Lower back (7) | 3 (2.4) | 0 (0.0) | 1 (1.5) | 2 (3.5) | 0.7487 |
| Abdomen (8) | 0 (0) | 0 (0.0) | 0 (0.0) | 0 (0.0) | - |
| Chest (9) | 1 (0.8) | 0 (0.0) | 0 (0.0) | 1 (1.8) | 0.5481 |
| Mid/ upper back (10) | 3 (2.4) | 0 (0.0) | 1 (1.5) | 2 (3.5) | 0.7487 |
| Neck (11) | 3 (2.4) | 0 (0.0) | 1 (1.5) | 2 (3.5) | 0.7487 |
| Head (12) | 0 (0) | 0 (0.0) | 0 (0.0) | 0 (0.0) | - |
| Shoulder (13) | 9 (7.2) | 1 (33.3) | 5 (7.7) | 3 (5.3) | 0.1818 |
| Forearm (14) | 2 (1.6) | 0 (0.0) | 2 (3.1) | 0 (0.0) | 0.3914 |
| Wrist (15) | 15 (12) | 1 (33.3) | 8 (12.3) | 6 (10.5) | 0.4926 |
| Finger (16) | 16 (12.8) | 1 (33.3) | 7 (10.8) | 8 (14.0) | 0.484 |
| **Duration of injury/ies, n (%)** |  |  |  |  |  |
| Less than 1 day (1) | 0 (0) | 0 (0.0) | 0 (0.0) | 0 (0.0) | 0.799 |
| 1-3 days (2) | 1 (2.3) | 0 (0.0) | 1 (4.3) | 0 (0.0) |  |
| 4-7 days (3) | 1 (2.3) | 0 (0.0) | 0 (0.0) | 1 (5.0) |  |
| 8-28 days (4) | 13 (29.5) | 0 (0.0) | 8 (34.8) | 5 (25.0) |  |
| 28 or more days (5) | 29 (65.9) | 1 (100) | 14 (60.9) | 14 (70.0) |  |
| **Who diagnosed the injury, n (%)** |  |  |  |  |  |
| Physician (1) | 7 (15.9) | 1 (100) | 3 (13.0) | 3 (15.0) | 0.4712 |
| PT (2) | 5 (11.4) | 0 (0.0) | 3 (13.0) | 2 (10.0) |  |
| Sports medicine specialist (Non-MD) (5) | 13 (29.5) | 0 (0.0) | 6 (26.1) | 7 (35.0) |  |
| Other health professional (6) | 3 (6.8) | 0 (0.0) | 3 (13.0) | 0 (0.0) |  |
| Climbing coach (7) | 1 (2.3) | 0 (0.0) | 0 (0.0) | 1 (5.0) |  |
| Myself (Self-diagnosed) (12) | 15 (34.1) | 0 (0.0) | 8 (34.8) | 7 (35.0) |  |
| **Did you stop training as a result of the injury, n (%)** |  |  |  |  |  |
| Yes (1) | 16 (36.4) | 1 (100) | 8 (34.8) | 7 (35.0) | 0.6365 |
| No (2) | 11 (25.0) | 0 (0.0) | 7 (30.4) | 4 (20.0) |  |
| Trained at reduced level (3) | 17 (38.6) | 0 (0.0) | 8 (34.8) | 9 (45.0) |  |
